# Evaluation of three rapid low-resource molecular tests for Nipah virus

**DOI:** 10.3389/fmicb.2022.1101914

**Published:** 2023-02-09

**Authors:** Nina M. Pollak, Malin Olsson, Glenn A. Marsh, Joanne Macdonald, David McMillan

**Affiliations:** ^1^Centre for Bioinnovation, University of the Sunshine Coast, Sippy Downs, QLD, Australia; ^2^DMTC Limited, Kew, VIC, Australia; ^3^School of Science, Technology and Engineering, University of the Sunshine Coast, Sippy Downs, QLD, Australia; ^4^Commonwealth Scientific and Industrial Research Organisation Health and Biosecurity, Australian Centre for Disease Preparedness, Geelong, VIC, Australia; ^5^BioCifer Pty Ltd., Brisbane, QLD, Australia

**Keywords:** Nipah virus (NiV), rapid test, isothermal amplification, nucleic acid lateral flow, point of care, recombinase polymerase amplification (RPA), recombinase-aided amplification (RAA), nucleic acid extraction

## Abstract

Accurate and timely diagnosis of Nipah virus (NiV) requires rapid, inexpensive, and robust diagnostic tests to control spread of disease. Current state of the art technologies are slow and require laboratory infrastructure that may not be available in all endemic settings. Here we report the development and comparison of three rapid NiV molecular diagnostic tests based on reverse transcription recombinase-based isothermal amplification coupled with lateral flow detection. These tests include a simple and fast one-step sample processing step that inactivates the BSL-4 pathogen, enabling safe testing without the need for multi-step RNA purification. The rapid NiV tests targeted the Nucleocapsid protein (N) gene with analytical sensitivity down to 1,000 copies/μL for synthetic NiV RNA and did not cross-react with RNA of other flaviviruses or Chikungunya virus, which can clinically present with similar febrile symptoms. Two tests detected 50,000–100,000 TCID_50_/mL (100–200 RNA copies/reaction) of the two distinct strains of NiV, Bangladesh (NiV_B_) and Malaysia (NiV_M_), and took 30 min from sample to result, suggesting these tests are well suited for rapid diagnosis under resource-limited conditions due to rapidity, simplicity, and low equipment requirements. These Nipah tests represent a first step toward development of near-patient NiV diagnostics that are appropriately sensitive for first-line screening, sufficiently robust for a range of peripheral settings, with potential to be safely performed outside of biohazard containment facilities.

## 1. Introduction

Nipah virus (NiV) is a zoonotic pathogen of the *Henipavirus* genus causing encephalitis and respiratory symptoms in humans with fatality rates of up to 75% (Eaton et al., 2006). Together with Hendra virus (HeV), they are the only paramyxoviruses that are classified as biosafety level 4 (BSL-4) pathogens. NiV has an exceptionally broad species tropism (Pernet et al., 2012). Transmission to humans can occur directly from *Pteropid* fruit bats (the reservoir host) or from contact with infected pigs (Luby et al., 2009; Clayton, 2017; Gurley et al., 2017), but also contaminated food. Interhuman transmission and nosocomial infections also contribute to Nipah dissemination (Weber and Rutala, 2001; Cleri et al., 2006; Sazzad et al., 2013). Cross-species transmissions of NiV have been reported as the causes of outbreaks in multiple South and Southeast Asia regions including Malaysia (Goh et al., 2000; Parashar et al., 2000), Singapore (Paton et al., 1999), India (Chadha et al., 2006; Arunkumar et al., 2019; Yadav et al., 2022), and Bangladesh (Sazzad et al., 2013). The range of the reservoir hosts has confined henipavirus spillover events to Asia and Australia, but detection of cross-reactive henipavirus antibodies in African bats (including West African fruit bats *Eidolon helvum*) and humans (Drexler et al., 2009; Pernet et al., 2014) markedly increased the number of people worldwide that live in regions at risk of henipavirus spillover events. There is no specific antiviral treatment for NiV infection, however, immunotherapeutic treatments (monoclonal antibody therapies) are currently under development (Playford et al., 2020). Nipah disease has been identified by WHO as a priority disease that poses a great public health risk due to its epidemic potential (World Health Organization [WHO], 2018).

Diagnostic tests with high sensitivity and specificity that enable early detection of NiV infection in humans are needed both for patient treatment and NiV disease control (Weber and Rutala, 2001; Cleri et al., 2006; Ahmed et al., 2009; Luby et al., 2009). Accurate diagnosis of NiV has traditionally relied on serological, molecular or virological analyses, which include western blotting, ELISA, plaque assay, immunofluorescence staining, genome detection by PCR and quantitative PCR, and virus isolation (Chua et al., 2000; Crameri et al., 2002; Drosten et al., 2003; Guillaume et al., 2004; Kashiwazaki et al., 2004; Wacharapluesadee and Hemachudha, 2007; Chiang et al., 2010; Kaku et al., 2012; Kulkarni et al., 2016; Fischer et al., 2018; Jensen et al., 2018; Schulz et al., 2020). Nucleic acid amplification tests (NAATs), such as reverse-transcriptase PCR (RT-PCR), are preferred for detection of active viral infection as they are highly sensitive and detect virus earlier in the infection cycle. However, the required laboratory infrastructure for NAATs may not be available in all endemic settings. For low-resource settings, isothermal amplification technologies offer highly sensitive and specific diagnosis of infectious diseases (Zhao et al., 2015; Zamani et al., 2021; Zheng et al., 2021). Combined with lateral flow detection (LFD), isothermal amplification offers a simple to use assay format that uses minimal equipment and is ideal for diagnostic point-of-care (POC) testing in resource-limited settings (James et al., 2018; Sadeghi et al., 2021; Ahmed et al., 2022). Of these, recombinase polymerase amplification (RPA) and recombinase-aided amplification (RAA) are two promising isothermal technologies. In RPA and RAA, double stranded DNA denaturation and strand invasion that is typically achieved by heat cycling in PCR is instead accomplished by a cocktail of recombinase enzymes, single-stranded binding proteins, and DNA polymerases (Piepenburg et al., 2006). Both methods have potential advantages over other technologies, such as loop-mediated isothermal amplification (LAMP), as they can be performed at near ambient temperature (37–42°C), are more rapid, require a less complex oligonucleotide design, and have higher tolerance to PCR inhibitors (Li et al., 2018, 2020).

Most isothermal approaches still require multiple nucleic acid extraction steps and/or two amplification steps to achieve high specificity (Zamani et al., 2021; Zheng et al., 2021). These additional steps increase workflow complexity, removing many of the benefits of the isothermal amplification for low-resource detection. Here we present a simple one-step method for NiV sample processing that requires only a single subsequent dilution step to enable processed samples to be directly used for nucleic acid amplification. The method uses a novel sample preparation reagent, TNA-Cifer Reagent E (BioCifer, Brisbane, QLD, Australia), which has been shown to process and inactivate dengue virus samples (Pollak et al., 2023). In this study, we trialed TNA-Cifer Reagent E for inactivation of NiV, and also trialed sample preparation testing in conjunction with three Nipah NAAT tests that were developed and characterized as part of our study. The three tests included RAA (Qitian, Jiangsu, China) and two formats of RPA (TwistDX, Cambridge, United Kingdom): the TwistAmp^®^ nfo kit and the TwistAmp^®^ exo kit (Li et al., 2018, 2020). Each test was trialed with LFD to provide a simple, low-resource results read-out. Our simple test format could improve the speed and ease of NiV point-of-care detection, providing vastly simplified workflows and improved laboratory safety that are greatly compatible with operation in low-resource settings.

## 2. Materials and methods

### 2.1. Plasmids, RNA transcripts and oligonucleotides

#### 2.1.1. Plasmids

Plasmids (pBIC-A) containing a NiV Nucleocapsid protein (N) gene fragment (JN808863.1, 694-993 nt) and a HeV N gene fragment (MN062017.1, 694-993 nt) were obtained from Bioneer Pacific (Victoria, Australia).

#### 2.1.2. RNA transcripts

Plasmids were linearized by restriction with Xho1 [New England Biolabs (Australia) Pty Ltd., Victoria, Australia], electrophoresed and purified (NucleoSpin^®^ Gel and PCR Clean-up, Macherey-Nagel, Düren, Germany). RNA transcripts were generated by *in vitro* transcription according to manufacturer’s instructions (MEGAscript^®^ T7 transcription kit, Invitrogen by Thermo Fisher Scientific Australia Pty Ltd., Victoria, Australia) and RNA concentration was determined with a Qubit 4 Fluorometer (Invitrogen by Thermo Fisher Scientific Australia Pty Ltd., Victoria, Australia).

#### 2.1.3. Oligonucleotides

Primers and probes for the recombinase-based isothermal amplification tests were designed from a consensus sequence of the highly conserved Nucleocapsid protein (N) gene coding region. A total of 100 published N gene sequences were first aligned to identify conserved regions within the gene. Primers and probes targeting these conserved regions were designed according to criteria described by the manufacturer (TwistDX). Primer-BLAST of NCBI was used to confirm the specificity of the primers and probes. The online OligoEvaluator software^1^ was used to analyze the potential for primer dimers and hairpins. The primers (5′ biotin labeled reverse) and probes [5′ 6-FAM (fluorescein) labeled] were synthesized by Bioneer Pacific (Victoria, Australia) using HPLC and PAGE purification, respectively. A total of three forward and three reverse primers, as well as two probes were synthesized, and tested in various combinations using reverse transcribed RNA as template and the optimized combination used for testing. Optimized sequences are in Table 1.

**TABLE 1:** Primer and probe sequences for the rapid Nipah virus (NiV) tests.

Name	Sequence
NiV F6	ATTCTTCGCAACCATCAGATTYGGGTTGGAG
NiV P2	[5′ Biotin] ATTCCAGAGTGACCTCAACACCATCAARAGC [Internal dS spacer] TGATGCTACTCTACAG [3′ C3 spacer]
NiV R5^a^	[5′ FAM] TCAAGAAGCACCATATAAGGGGCTCTTGGG
NiV R6^b^	[5′ FAM] TTAGTCTGAATTGATTCTTCAAGAAGCACC

^a^RT-RPA_NFO_ and RT-RPA_EXO_.

^b^RT-RAA.

### 2.2. Viruses

#### 2.2.1. Virus strains

NiV strains were originally derived from two human isolates, Nipah/Bangladesh/Human/2004/Rajbari, R1 (GenBank accession no. AY988601; NiV_B_) and Nipah virus/Malaysia/Human/99 (GenBank accession no. AF212302; NiV_M_). Hendra virus was originally isolated from the lung of a horse (Hendra virus/Australia/Horse/2008/Redlands; GenBank accession no. HM044317; HeV). For inactivation, virus stocks were sent to a commercial gamma-irradiator where they were treated with 50 kGy gamma-irradiation. Flavivirus strains were originally derived from clinical isolates included Dengue virus serotypes 1–4 (DENV1 ET00.243 GenBank accession no. JN415499, DENV-2 ET00.300 GenBank accession no. JN568254, DENV-3 East Timor 2000 GenBank accession no. JN575566, and DENV-4 ET00.288 GenBank accession no. JN575585), Japanese encephalitis virus (JEV Nakayama strain GenBank accession no. EF571853), West Nile virus Kunjin strain (WNV_KUNV_ NSW2011 strain GenBank accession no. JN887352), Murray Valley encephalitis virus (MVEV 1-51 strain GenBank accession no. L48972), and Zika virus (ZIKV MR766 GenBank accession no. MW143022). Alphavirus strain originally derived from clinical isolate Chikungunya virus Mauritius 2006 (CHIKV GenBank accession no. MH229986).

#### 2.2.2. Cell culture

Vero cells (Vero C1008) were obtained from ATCC. Vero cells were grown in Minimal Essential Medium (Gibco by Thermo Fisher Scientific Australia Pty Ltd., Victoria, Australia) containing 1× Antibiotic/Antimycotic solution (Gibco by Thermo Fisher Scientific Australia Pty Ltd., Victoria, Australia), and 10% fetal calf serum (Gibco by Thermo Fisher Scientific Australia Pty Ltd., Victoria, Australia), designated MEM-10), at 37°C and 5% CO_2_. *Aedes albopictus* clone C6/36 (ATCC CRL-1660) were obtained from the American Type Culture Collection. C6/36 cells were cultured in RPMI 1640 (Thermo Fisher Scientific Australia Pty Ltd., Victoria, Australia) with 5% heat−inactivated fetal bovine serum (Sigma-Aldrich, New South Wales, Australia), 2 mmol/L L−glutamine (Gibco by Thermo Fisher Scientific Australia Pty Ltd., Victoria, Australia) and 100 U/mL Penicillin, 100 μg/mL Streptomycin and 0.25 μg/mL Amphotericin B (Sigma-Aldrich, New South Wales, Australia), at 28°C and 5% CO_2_. Before reaching confluency, Vero and C6/36 cells were trypsinized with 0.25% trypsin solution (Gibco by Thermo Fisher Scientific Australia Pty Ltd., Victoria, Australia), and resuspended in fresh growth media before plating onto a new growth surface.

#### 2.2.3. Virus culture

Hendra virus and NiV were propagated in Vero cells with a low MOI infection of T175 cm flask. Virus containing supernatant was harvested at approximately 72 h, when significant cytopathic effect (CPE) was visible, stock clarified by centrifugation at 5,000 g for 10 min and then stored at −80°C until needed. NiV_B_, NiV_M_, and HeV stock titers were 5.54 × 10^7^ tissue culture infectious dose (TCID)_50_/mL, 1.89 × 10^7^ TCID_50_/mL, and 9.5 × 10^7^ TCID_50_/mL. Flavivirus strains and CHIKV were propagated to a concentration of 10^5^–10^7^ TCID_50_/mL in T25 culture flasks seeded with C6/36 cells in RPMI 1,640 growth media as described above, with the exception that 2% FBS was used. Seven days post infection, 2 mL of TRI Reagent (Sigma-Aldrich, New South Wales, Australia) was added to the flask preparation and swirled over the cell area for 1–2 min. To prepare the inactivated viruses for extraction the inoculum was separated into a tube and centrifuged at 3,000 rpm for 10 min at 4°C to separate supernatant from cell pellet. The total RNA for all flavivirus strains and CHIKV was extracted from the infected culture cell lysate stocks using the TRI Reagent extraction protocol, resuspended in nuclease-free water, quantified using a NanoDrop 2000 spectrophotometer (Thermo Fisher Scientific Australia Pty Ltd., Victoria, Australia) and stored at −80°C.

### 2.3. NiV and HeV RNA isolation and TaqMan PCR

RNA was extracted from NiV and HeV stocks using a MagMAX™ Viral RNA Isolation Kit (ThermoFisher) following manufacturer’s instructions. RNA was eluted in a final volume of 60 μL. Samples were stored at −80°C prior to Taqman PCR analyses. TaqMan qPCR was performed using the AgPath-ID one-step reverse transcription-PCR kit (ThermoFisher), targeting the N gene of HeV or NiV as previously described (Feldman et al., 2009). Copy numbers were calculated using a previously derived formula from a standard curve.

### 2.4. Rapid NiV tests

#### 2.4.1. NiV assay design

The rapid, low-resource NiV tests targeted the Nucleocapsid protein (N) gene of NiV (nt 910-1056 for RT-RPA_NFO_ and RT-RPA_EXO_, nt 910-1047 for RT-RAA), as this region has previously been used for development of qPCR assays (Jensen et al., 2018). By analyzing multiple sequence alignments of NiV strains of the Malaysian and Bangladesh genotypes, a highly conserved region was chosen for primer and probe design suitable for use in recombinase-based isothermal amplification reactions adhering to the general rules for RPA primer and probe design provided by TwistDx (2018). Different combinations of forward and reverse primers, and probes were tested using reverse transcribed RNA as template to identify an optimized combination used for all further testing.

#### 2.4.2. Sample processing

Gamma-irradiated NiV_B_, NiV_M_, and HeV isolates diluted in viral transport medium (VTM; Minimal Essential Medium containing 0.1% bovine serum albumin (Thermo Fisher Scientific Australia Pty Ltd., Victoria, Australia), 500 U/mL Penicillin (Gibco by Thermo Fisher Scientific Australia Pty Ltd., VIC, Australia), 500 μg/mL Streptomycin (Gibco by Thermo Fisher Scientific Australia Pty Ltd., Victoria, Australia) and 2,500 μg/mL Fungizone (Gibco by Thermo Fisher Scientific Australia Pty Ltd., Victoria, Australia) were mixed with TNA-Cifer Reagent E (BioCifer, Brisbane, QLD, Australia) at a ratio of 1:1 (4 μL sample to 4 μL TNA-Cifer Reagent E) and incubated for 2 min on ice. Processed samples were then diluted 1:6 (5 μL processed sample to 25 μL nuclease-free water) and immediately used as a template for either RT-nfoRPA (section “2.4.3 NiV RT-nfoRPA or RT-exoRPA tests”), RT-exoRPA (section “2.4.3 NiV RT-nfoRPA or RT-exoRPA tests”), or RT-RAA (section “2.4.4 NiV RT-RAA tests”) reaction without further RNA purification.

#### 2.4.3. NiV RT-nfoRPA or RT-exoRPA tests

Each test reaction was prepared using our developed primers and probe combined with either the TwistAmp™ nfo kit (TwistDX, Cambridge, United Kingdom) or the TwistAmp™ exo kit (TwistDX, Cambridge, United Kingdom), with final reaction conditions of 1× rehydration buffer and 1/5 rehydrated lyophilized pellet, forward primer (420 nM), reverse primer (420 nM), probe (120 nM), Ribolock (10 U), and Moloney Murine Leukemia virus reverse transcriptase (mMLV, 40 U) including 1 μL template (from section “2.4.2 Sample processing”) and magnesium acetate (14 mM) to a final reaction volume of 10 μL. Reactions using the TwistAmp™ exo kit also contained Endonuclease IV (2 U; New England Biolabs, Victoria, Australia). Reactions were incubated at 39°C for 20 min before lateral flow detection.

#### 2.4.4. NiV RT-RAA tests

Each test reaction was prepared using our developed primers and probe combined with the RAA kit (Qitian, Jiangsu, China), with final reaction conditions of 1.224× rehydration buffer and 1/5 rehydrated lyophilized pellet, forward primer (420 nM), reverse primer (420 nM), probe (240 nM), Moloney Murine Leukemia virus reverse transcriptase (mMLV, 40 U; Promega, New South Wales, Australia), SuperScriptIV (40 U; Invitrogen, Victoria, Australia), RNase H (0.4 U; Invitrogen, Victoria, Australia) and Endonuclease IV (2 U; New England Biolabs, Victoria, Australia) including 1 μL template (from section “2.4.2 Sample processing”) and magnesium acetate (14 mM) to a final reaction volume of 10 μL. Reactions were incubated at 39°C for 20 min before lateral flow detection.

#### 2.4.5. Lateral flow detection and analysis

Two microliters of the amplified test reaction mix (from sections “2.4.3 NiV RT-nfoRPA or RT-exoRPA tests or 2.4.4 NiV RT-RAA tests”) was added to pre-activated HybriDetect lateral flow strips (Milenia Biotec, Giessen, Germany) (Rames and Macdonald, 2019), a universal dipstick for the detection of biotin- and fluorescein (FITC or FAM)-labeled analytes based on lateral flow technology using gold particles. The strips were then placed for 5 min in 100 μL running buffer (Li et al., 2019), analyzed by eye and scanned with an Epson Perfection V39 Flatbed Scanner (Epson, NSouth Wales, Australia). On visual analysis, a single control line depicted the absence of NiV and the appearance of two lines i.e., a test line along with the control line indicated the presence of NiV. Lateral flow strips were analyzed as previously described (James et al., 2018; Li et al., 2019) using ImageJ software (National Institutes of Health, MD, USA).

### 2.5. Statistical data analyses

Diagnostic test evaluation and comparison was determined using the online MedCalc statistical software.^2^

## 3. Results

### 3.1. NiV isothermal assay analytical sensitivity

**FIGURE 1:**
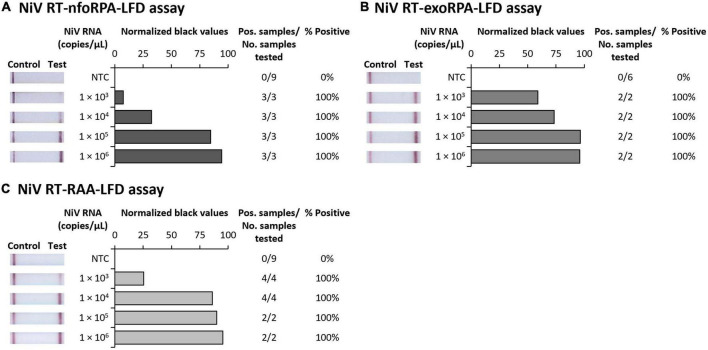
Analytical sensitivities of three Nipah virus recombinase-based isothermal amplification lateral flow detection assays with RNA transcripts. Sensitivity testing used Nucleoprotein (N) gene fragment RNA transcripts diluted 10-fold in water for RT-nfoRPA-LFD **(A)**, RT-exoRPA-LFD **(B)**, and RT-RAA-LFD **(C)**. Images of lateral flow strips with two bands (control and test band) indicates the sample is positive for NiV synthetic RNA transcript, and single control band indicates a valid reaction with negative sample. Photograph of lateral flow strips with control bands (all samples) and test bands (positive samples) compared to copy number of serially diluted NiV RNA (copies/μL) and no template control (NTC) (left). Normalized pixel density (normalized black values) from the lateral flow test strip displayed (middle). Positive samples compared to number of samples tested at that dilution was used to calculate the percentage of positive tests performed at that dilution (right).

To test the analytical sensitivity of the three NiV assays (NiV RT-nfoRPA-LFD, RT-exoRPA-LFD, and RT-RAA-LFD), serial dilutions of a synthetic template RNA with known copy number were assessed. The analytical sensitivity ranged from the highest concentration tested (1 × 10^6^ copies/μL) to as little as 1,000 copies/μL for all three tests (Figures 1A–C).

### 3.2. NiV isothermal assay analytical specificity

**FIGURE 2:**
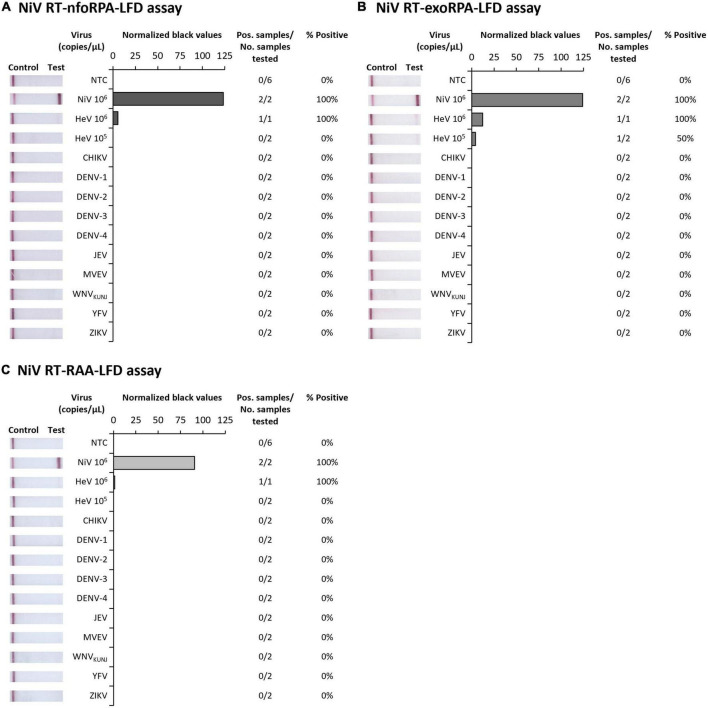
Analytical specificities of three Nipah virus recombinase-based isothermal amplification lateral flow detection tests. Specificity testing used synthetic RNA of NiV and Hendra virus (HeV), and viral RNA extracts from alphavirus Chikungunya virus (CHIKV), and flaviviruses DENV-1, DENV-2, DENV-3, DENV-4, JEV, MVEV, WNV_KUNJ_, YFV, and ZIKV for RT-nfoRPA-LFD **(A)**, RT-exoRPA-LFD **(B)**, and RT-RAA-LFD **(C)**. Images of lateral flow strips with two bands (control and test band) indicates the sample is positive for respective viral RNA extract, and single control band indicates a valid reaction with negative sample. Nuclease-free water was tested as the no template control (NTC; left). Normalized pixel density (normalized black values) from the test displayed (middle). Positive samples compared to number of samples tested using different viral RNA transcripts and extracts were used to calculate percentage of positive samples (right).

Since symptoms of NiV infection are similar to other febrile diseases, specific diagnosis is critical for containment of an outbreak and to facilitate appropriate patient care. To confirm our NiV assays were specific for NiV, we next trialed our three tests against synthetic HeV RNA transcripts and RNA extracts from Chikungunya virus (CHIKV), Dengue virus serotypes 1-4 (DENV 1-4), Japanese encephalitis virus (JEV), Murray Valley encephalitis virus (MVEV), West Nile virus Kunjin strain (WNV_*KUNJ*_), Yellow fever virus (YFV), and Zika virus (ZIKV). Our Nipah tests did not detect CHIKV or any flaviviruses, however, HeV was detected at very high concentrations (10^6^ copies/μL) showing faint positive test bands (Figures 2A–C). Testing HeV at lower concentration (10^5^ copies/μL) revealed no positive test result with the RT-nfoRPA-LFD and RT-RAA-LFD. The RT-exoRPA-LFD resulted in a positive test result in one out of two replicates, suggesting more pronounced non-specific detection of synthetic HeV RNA compared to the other two test formats.

### 3.3. Inactivation of NiV using TNA-Cifer Reagent E

**FIGURE 3:**
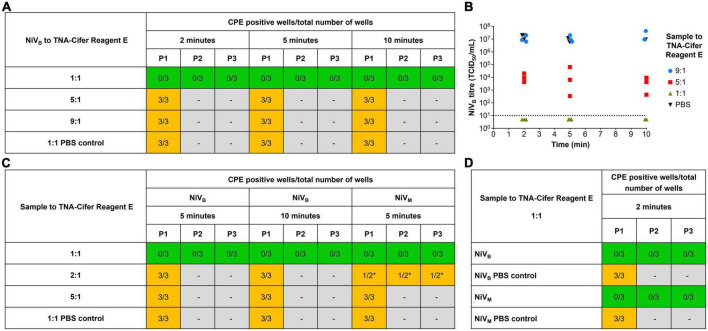
Rapid sample processing inactivates Nipah virus. **(A)** NiV_B_ recovery from samples (5.54 × 10^7^ TCID_50_/mL) incubated with and without TNA-Cifer Reagent E at a 1:1, 5:1 and 9:1 ratio, for 2, 5 or 10 min at room temperature. **(B)** NiV_B_ was incubated with TNA-Cifer Reagent E at 1:1, 5:1 and 9:1 ratio or PBS at 9:1 for the indicated times at room temperature and the virus titer determined by TCID_50_ assays using Vero E6 cells. The dotted line indicates limit of detection of assays based on a starting 1/10 dilution of samples. This experiment was performed one time, with three samples taken per time point. **(C)** NiV recovery from samples (NiV_B_ 5.54 × 10^7^ TCID_50_/mL; NiV_M_ 1.89 × 10^7^ TCID_50_/mL) incubated with and without TNA-Cifer Reagent E at a 1:1, 2:1 and 5:1 ratio for 5 and/or 10 min at room temperature. **(D)** NiV recovery from samples (NiV_B_ 5.54 × 10^7^ TCID_50_/mL; NiV_M_ 1.89 × 10^7^ TCID_50_/mL) incubated with and without TNA-Cifer Reagent E at a 1:1 ratio and 2 min at room temperature. **(A, C, D)** Mixtures (100 μL) were added to individual wells of Vero E6 cells (approximately 70% confluent) on 6-well plate and incubated for 7 days. Individual wells were scored as either positive or negative for the presence of cytopathic effect (CPE) typical of Nipah virus (syncytia). Wells showing no evidence of NiV CPE had 200 μL supernatant removed and added to a new 6-well plate with fresh Vero cells (blind passage), again incubated for 7 days before scoring for NiV CPE. This blind passaging of negative samples was done for two additional passages (three 7 days incubations on Vero cells: P1, P2, and P3). Number of CPE positive wells compared to total number of wells for each condition. Green highlighted boxes show samples that were inactivated by TNA-Cifer Reagent E and showed no CPE. Orange highlighted boxes indicate samples that were not inactivated and showed CPE. Gray highlighted boxes mark where samples were not passaged due to the presence of CPE in the previous passage. Each experiment was performed in triplicate, except were indicated by * the experiment was performed in duplicate due to human error.

To develop a truly low-resource test, we wanted to trial the NAAT tests in conjunction with sample preparation using TNA-Cifer Reagent E. However, since NiV is a BSL-4 agent, we first had to determine the conditions in which TNA-Cifer Reagent E would inactivate NiV, before any processed samples could be used for NAAT detection. TNA-Cifer Reagent E was found to completely inactivate NiV_B_ (5.54 × 10^7^ TCID_50_/mL) in 2 min after mixing the sample and reagent in a 1:1 ratio, and this was confirmed by serial passaging of inactivated virus for a further two serial passages at 2, 5, and 10 min (Figure 3A). However, sample to reagent ratios of 5:1 and 9:1 failed to inactivate NiV_B_ during any of the tested incubation periods (2, 5, and 10 min, Figure 3B), and subsequent testing with NiV_B_ and NiV_M_ demonstrated that sample to reagent ratios of 2:1 also failed after both 5 and 10 min incubation (Figure 3C). These results suggested that equal parts of sample and TNA-Cifer Reagent E were required for successful NiV inactivation. Further investigation confirmed complete inactivation of both NiV_B_ (5.54 × 10^7^ TCID_50_/mL) and NiV_M_ (1.89 × 10^7^ TCID_50_/mL) at a 1:1 ratio after 2 min incubation, and this was confirmed by serially passaging culture supernatant through a further two serial passages (Figure 3D).

### 3.4. Sensitivity and specificity of rapid NiV tests using rapidly processed gamma-irradiated henipavirus isolate samples

**FIGURE 4:**
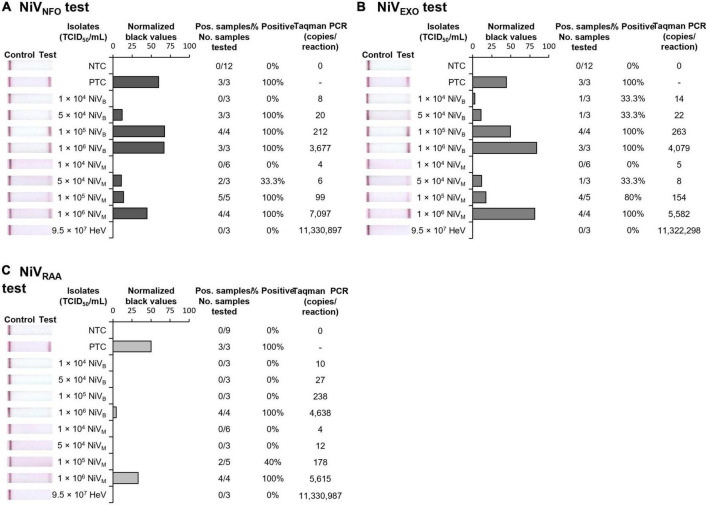
Sensitivity and specificity of three rapid NiV tests. Sensitivity testing used rapidly processed NiV_B_ and NiV_M_ strain isolates for RT-nfoRPA-LFD **(A)**, RT-exoRPA-LFD **(B)**, and RT-RAA-LFD **(C)** assays compared to TCID_50_/mL determined by immunolabeling assays and copies/reaction determined by comparative Taqman PCR. Images of lateral flow strips with two bands (control and test band) indicates the sample is positive for NiV, and single control band indicates a valid reaction with negative sample. Photograph of lateral flow strips with control bands (all samples) and test bands (positive samples) compared to titer of rapidly processed serially diluted NiV isolate (TCID_50_/mL) and no template control (NTC) (left). Normalized pixel density (normalized black values) from the lateral flow test strip displayed (middle). Positive samples compared to number of samples tested at that dilution was used to calculate the percentage of positive tests performed at that dilution (right).

By combining the rapid sample preparation method with our three isothermal NiV assays, we were able to develop three rapid NiV tests able to provide results in 30 min from sample to results. The rapid NiV_NFO_, rapid NiV_EXO_, and rapid NiV_RAA_ tests combined rapid sample preparation followed by RT-nfoRPA-LFD, RT-exoRPA-LFD, or RT-RAA-LFD, respectively. To assess strain-specific sensitivities, two distinct strains of gamma-irradiated NiV isolates, Bangladesh (NiV_B_), and Malaysia (NiV_M_), were serially diluted in viral transport medium and tested with each rapid NiV test. Using this approach, we detected both NiV_B_ and NiV_M_ with two of the three rapid NiV tests (NiV_NFO_ and NiV_EXO_) in the range of 50,000–100,000 TCID_50_/mL, the equivalent of approximately 50–100 infectious particles per microliter (Figures 4A, B). However, the rapid NiV_RAA_ test was shown to detect virus at only 1,000,000 TCID_50_/mL (Figure 4C). All three NiV tests did not detect HeV at very high concentrations (95,000,000 TCID_50_/mL).

Combining all gamma-irradiated virus testing results from Figure 4 together, we assessed the diagnostic sensitivity and specificity of the test, for detection of henipavirus isolate samples at 100,000 TCID_50_/mL or higher (*n* = 25, Table 2). The best performing test, the rapid NiV_NFO_ test, had 100.0% diagnostic sensitivity (95% CI: 79.4–100.0%) and 100.0% diagnostic specificity (95% CI: 29.24–100.0%). The other two Rapid NiV tests (NiV_EXO_ and NiV_RAA_) demonstrated diagnostic sensitivities of 93.8% (95% CI: 69.8–99.8%) and 62.5% (95% CI: 35.4–84.8%), respectively, with both showing 100% diagnostic specificity (95% CI: 29.2–100.0%) (*n* = 25, Table 2).

**TABLE 2:** Diagnostic test evolution of three rapid Nipah virus (NiV) tests.

Rapid NiV test	#True Pos.	True Neg.	False Pos.	False Neg.	Total tested	Sensitivity (95% CI)	Specificity (95% CI)
RT-nfoRPA-LFD	16	3	0	0	19	100.0% (79.4–100.0%)	100.0% (29.2–100.0%)
RT-exoRPA-LFD	15	3	0	1	19	93.8% (69.8–99.8%)	100.0% (29.2–100.0%)
RT-RAA-LFD	10	3	0	6	19	62.5% (35.4–84.8%)	100.0% (29.2–100.0%)

Conservative analysis included all tested henipavirus isolate samples ≥100,000 TCID_50_/mL.

## 4. Discussion

Given the often rural and remote NiV outbreak settings, NiV diagnostics should ideally be deployable for use in decentralized laboratories or field-based settings, and at the same time, still fulfill the need for sensitive and accurate detection of early NiV infection. Isothermal NAAT platforms have lower infrastructure requirements than laboratory-based diagnostics. If these could be combined with safe and simple sample preparation methods, they could be deployable for low-resource use, with fewer training requirements for healthcare workers. In this study, we developed and evaluated three rapid NiV tests in a simple low-resource format. These tests use a simple and rapid sample preparation protocol, a single-temperature isothermal amplification technology (RT-nfoRPA, RT-exoRPA, or RT-RAA), and are coupled with LFD for easy result interpretation within 30 min. The tests were shown to be specific for detection of NiV, and did not detect other viruses which can clinically present with similar febrile symptoms including flaviviruses, CHIKV. For the closely related HeV we note that synthetic HeV transcript was detected at very high concentrations (10^6^ copies/μL), but gamma-irradiated HeV could not be detected at very high titers (9.5 × 10^7^ TCID_50_/mL) suggesting that the assays were specific for NiV. The tests can be used without expensive laboratory equipment, and could prevent extended waiting periods for NiV testing, by enabling on-site low-resource testing rather than delayed results due to sample shipment to central laboratory testing facilities.

The majority of previous studies have used RT-qPCR for the detection of NiV nucleic acids. Only one study has reported the development of an RT-LAMP assay targeting the N gene capable of detection of 100 pg (estimated approximately 10^7^ copies/reaction) of total Nipah pseudovirus RNA, suggesting its detection limit was comparable to conventional RT-qPCR (Ma et al., 2019). The reported assay tested RNA prepared with a viral nucleic acid extraction kit and produced results in either 45 or 50 min with a Realtime Turbidimeter or a calcein dye detection method with a water bath (Ma et al., 2019). In comparison, two of our three rapid NiV tests (rapid sample processing followed by RT-nfoRPA-LFD or RT-exoRPA-LFD) had a detection limit ranging from 50,000 to 100,000 TCID_50_/mL, equivalent to 20–263 infectious particles per microliter, or 20–250 RNA copies/reaction. The best performing rapid NiV test (rapid sample processing combined with RT-nfoRPA-LFD) showed 100.0% diagnostic sensitivity (95% CI: 79.4–100.0%) and 100.0% diagnostic specificity (95% CI: 29.24–100.0%) for detection of >100,000 TCID_50_/mL virus (100–200 RNA copies/reaction). The rapid NiV test based on the exo product which required the addition of the enzyme endonucleases IV showed reduced diagnostic sensitivity (93.8, 95% CI: 69.8–99.8%; 200–250 RNA copies/reaction), but may represent a suitable alternative to the currently unavailable nfo product. In comparison, our rapid NiV test utilizing RAA kits only showed a diagnostic sensitivity of 62.5% (95% CI: 35.4–84.8%; 4,000–6,000 RNA copies/reaction), despite optimization which included slightly more rehydration buffer (1.224×) which slightly improved results. However, RT-RAA tests have previously been reported to reach high sensitivity (>95%) for the detection of SARS-CoV-2 (Qian et al., 2020; Subsoontorn et al., 2020).

Preparing samples for nucleic acid testing is a critical step for any field-based diagnostic POC method, particularly for NAATs that traditionally require high purity of samples. Diagnosis of suspected Nipah cases could greatly benefit from the elimination of traditional RNA extraction kit use, particularly where a lack of infrastructure exists and excludes the use of automated costly robotics systems. Previously reported in house NAATs employed for NiV detection used commercial RNA extraction reagents and kits [reviewed in Mazzola and Kelly-Cirino (2019)], which require time consuming procedures or costly automated robotic systems, and cannot be performed if access to centralized well-equipped laboratories including robots, centrifuges and/or vacuum manifolds, is limited. One key advantage of our rapid NiV tests was the unique sample processing method that enabled rapid virus detection in NiV isolates, while inactivating the BSL-4 pathogen in the first step of the procedure. Our sample processing method reduced sample preparation time to only 2 min, involving the direct addition of a single reagent to the sample followed by dilution in nuclease-free water before subsequent isothermal amplification and did not require time-consuming multistep RNA extraction with a kit. Our simple sample processing method can provide a pathway for safer POC testing, or safer near-patient POC testing in low-resource environments that do not have full biohazard facilities. Additional work is required to clinically validate the performance and operational utility of our rapid NiV tests. Validation efforts for NiV tests have been limited due to the lack of NiV-positive human samples. This study limitation could be addressed by spiking gamma-irradiated NiV into blood, plasma, and serum, in an effort to show operational suitability with clinical samples. In addition, further analysis of NiV inactivation should consider the minimal TNA-Cifer Reagent E concentration, as well as the effect of serum, sub-optimal sample matrices, or other potential inhibitors of inactivation before field trialing tests.

NiV has both epidemic and pandemic potential (Gomez Roman et al., 2020). The virus has been shown to transmit through contaminated food, as well as *via* direct contact with animal excretions or infected humans. Human NiV incubation periods after exposure are believed to range from 4 to 14 days, with reports as long as 45 days (Chong et al., 2002). It is unclear if transmission can occur during this time, but likely begins during the incubation period, which has been demonstrated in pigs (Middleton et al., 2002). Pigs and experimentally infected cats can shed NiV in respiratory secretions and urine (Middleton et al., 2002; Mungall et al., 2007). Many other viruses in the Paramyxoviridae family (like measles virus) transmit well between people, so there is concern that a novel Nipah variant with increased transmission could arise. Our rapid NiV test could be used to rapidly detect disease at or near-POC, to enable early interventions that can reduce morbidity and mortality. It would also be interesting to consider extending this rapid test format to detect all henipaviruses (NiVB/NiVM, HeV, and Cedar virus) or all paramyxoviruses to further improve detection of these pathogens at the POC.

In conclusion, we developed three rapid Nipah tests and evaluated their potential as near-patient POC or POC diagnostic suitable for resource-limited settings. We determined the analytical sensitivity of the three recombinase-based isothermal amplification LFD tests to be at least 1,000 copies/μL, and confirmed the tests did not detect any other viruses displaying similar febrile symptoms. The rapid NiV tests demonstrated excellent diagnostic specificity and varying degrees of sensitivity (62.5–100.0%) for detection of >100,000 TCID_50_/mL virus. The tests are advantageous compared to conventional methods such as RT-PCR, with improved procedural simplicity, rapid sample processing and turnaround time (30 min from sample preparation to result), minimal equipment requirements, and improved safety as the BSL-4 pathogen is inactivated in the very first step of the procedure. Our three Nipah tests represent a first step toward development of near-patient POC or POC NiV diagnostics that are affordable, appropriately sensitive for first-line screening, and sufficiently robust for adequate testing at the community level. Future research could focus on establishing operational suitability of the selected rapid NiV test, by integration of the whole testing process into a microfluidic system to facilitate a rapid, accurate, and safe testing procedure for testing at the POC level.

## Data availability statement

The original contributions presented in this study are included in this article/supplementary material, further inquiries can be directed to the corresponding authors.

## Author contributions

NP: conceptualization, data curation, formal analysis, investigation, methodology, project administration, resources, supervision, validation, visualization, writing—original draft preparation, and writing—review and editing. MO: data curation, formal analysis, investigation, methodology, validation, visualization, and writing—review and editing. GM: data curation, formal analysis, investigation, methodology, resources, validation, visualization, and writing—review and editing. JM: conceptualization, formal analysis, funding acquisition, methodology, project administration, resources, supervision, and writing—review and editing; DM: conceptualization, formal analysis, funding acquisition, methodology, project administration, resources, supervision, and writing—review and editing. All authors contributed to the article and approved the submitted version.

## Footnotes

1
http://www.oligoevaluator.com

2
https://www.medcalc.org/calc/diagnostic_test.php
